# Effect of Sodium-Glucose Cotransport Inhibition on Polycystic Kidney Disease Progression in PCK Rats

**DOI:** 10.1371/journal.pone.0125603

**Published:** 2015-04-30

**Authors:** Sarika Kapoor, Daniel Rodriguez, Meliana Riwanto, Ilka Edenhofer, Stephan Segerer, Katharyn Mitchell, Rudolf P. Wüthrich

**Affiliations:** 1 Division of Nephrology, University Hospital, Zürich, Switzerland; 2 Institute of Physiology, University of Zürich, Zürich, Switzerland; 3 Clinic for Equine Internal Medicine, Vetsuisse Faculty, University of Zürich, Zürich, Switzerland; University of Geneva, SWITZERLAND

## Abstract

The sodium-glucose-cotransporter-2 (SGLT2) inhibitor dapagliflozin (DAPA) induces glucosuria and osmotic diuresis via inhibition of renal glucose reabsorption. Since increased diuresis retards the progression of polycystic kidney disease (PKD), we investigated the effect of DAPA in the PCK rat model of PKD. DAPA (10 mg/kg/d) or vehicle was administered by gavage to 6 week old male PCK rats (n=9 per group). Renal function, albuminuria, kidney weight and cyst volume were assessed after 6 weeks of treatment. Treatment with DAPA markedly increased glucose excretion (23.6 ± 4.3 vs 0.3 ± 0.1 mmol/d) and urine output (57.3 ± 6.8 vs 19.3 ± 0.8 ml/d). DAPA-treated PCK rats had higher clearances for creatinine (3.1 ± 0.1 vs 2.6 ± 0.2 ml/min) and BUN (1.7 ± 0.1 vs 1.2 ± 0.1 ml/min) after 3 weeks, and developed a 4-fold increase in albuminuria. Ultrasound imaging and histological analysis revealed a higher cyst volume and a 23% higher total kidney weight after 6 weeks of DAPA treatment. At week 6 the renal cAMP content was similar between DAPA and vehicle, and staining for Ki67 did not reveal an increase in cell proliferation. In conclusion, the inhibition of glucose reabsorption with the SGLT2-specific inhibitor DAPA caused osmotic diuresis, hyperfiltration, albuminuria and an increase in cyst volume in PCK rats. The mechanisms which link glucosuria to hyperfiltration, albuminuria and enhanced cyst volume in PCK rats remain to be elucidated.

## Introduction

Polycystic kidney diseases (PKD) are the most frequent entities among the genetically determined renal syndromes [1]. The autosomal dominant form of PKD (ADPKD) is twenty times more frequent than the autosomal recessive form (ARPKD) [2]. Approximately 5–8% of all patients with end-stage renal disease (ESRD) suffer from ADPKD [3]. Although progress has recently been made in the development of treatments which retard the cystic growth, no therapy was shown to be effective in delaying the occurrence of ESRD [4].

It has been shown that renal cAMP is a major driver of cyst growth in PKD [5]. The excessive cAMP production is a consequence of the genetic defect which underlies PKD [6]. Due to an early loss of the urine concentrating capability the production of vasopressin is upregulated in PKD, stimulating the production of cAMP directly through its V_2_ receptor in the distal renal epithelium [7]. Therapeutic strategies which decrease the vasopressin-driven cAMP production have been successful in decreasing renal cyst growth and in slowing the decline of renal function in PKD [8–11]. Thus, treatment of mice, rats and humans with the vasopressin V_2_-receptor antagonist tolvaptan [12], crossing PKD rats (PCK strain) with vasopressin-deficient rats (Brattleboro strain) [13], or increasing fluid intake in rats by adding glucose to the drinking water [14] have all been effective to retard PKD disease progression. Patients with ADPKD tend to have a higher urine output because of a renal concentrating defect and a blunted release of vasopressin [15], but presumably also because drinking large amounts of water has been recommended to patients with ADPKD in an attempt to reduce cyst growth [16,17].

As mentioned, the aquaretic drug tolvaptan (vasopressin V_2_ receptor antagonist) was shown to have beneficial effects on polycystic kidney disease progression. It is not known whether the induction of osmotic diuresis would also have such a beneficial effect. We have previously shown that the induction of osmotic diuresis by inhibiting renal proximal tubular sodium-glucose cotransport (SGLT) with phlorizin retards cyst growth and renal functional decline in the Han:SPRD rat model of PKD [18]. Phlorizin is a nonselective SGLT inhibitor which inhibits SGLT1 and SGLT2. In recent years, selective SGLT2 inhibitors have been developed and are now in clinical use for the treatment of hyperglycemia in patients with type 2 diabetes mellitus [19]. To evaluate whether the selective inhibition of SGLT2 is capable of retarding cyst volume progression and delaying renal functional loss, we tested the effect of oral dapagliflozin (DAPA) administration in PCK rats, an orthologous model of ARPKD.

## Materials and Methods

### Ethics statement

All animal work was conducted according to relevant national and international guidelines. The protocol was approved by the committee on the Ethics of Animal Experiments at the University of Zürich (Permit Number: 175–2012). All efforts were made to minimize any suffering to animals.

### Animals

PCK rats (an orthologous model of autosomal recessive polycystic kidney disease) and normal Sprague-Dawley (SD) rats were used in this study. PCK rats (originally derived from SD rats) were obtained from Charles River Laboratories (Sulzfeld, Germany) while SD rats were obtained from the Rat Resource and Research Center (Columbia, MO, USA). All rats had free access to tap water and were fed a standard rat diet. Only male rats were used in this study as cysts develop more rapidly in male compared with female rats.

### Experimental design

DAPA (Bristol-Myers Squibb, Princeton, New Jersey) was dissolved in a vehicle of polypropylene glycol, water and ethanol (45:45:10, v/v/v). At 5–6 weeks of age, male PCK or normal control SD rats (n = 8–9 per group) were given DAPA (10 mg/kg/d) or vehicle by gavage for 5–6 weeks. The doses of the drug and the vehicle were adjusted daily according to the body weight of the rats. Blood was drawn and 24 h urine was collected at baseline and after 2–3 and 5–6 weeks of treatment to assess different parameters of renal function. At the end of the treatment phase (at 12 week of age), the PCK rats were examined by ultrasound to determine the kidney and cyst volume *in vivo*. All rats were then sacrificed and the kidneys were excised, decapsulated and weighed to calculate the two kidneys to total body weight (2K/TBW) ratios. Kidney slices were fixed in 10% buffered formalin and were then embedded in paraffin for subsequent histological examinations.

### Plasma and urine analyses

Plasma and urine aliquots were rapidly frozen and stored at -80°C until their measurement. Glucose, sodium, chloride, creatinine and blood urea nitrogen (BUN) concentrations were determined in plasma and urine using a Cobas 8000 Modular Analyzer from Roche Diagnostics AG (Rotkreuz, Switzerland). Plasma and urine osmolality were measured by using an Advanced Osmometer Model 2020 (Advanced Instruments Inc., Norwood, MA, USA). The urine protein content was analyzed by SDS-PAGE and Coomassie blue staining, adjusting the loading volumes of the samples to the 24 h urine volume. The GenWay rat albumin ELISA kit (San Diego, CA, USA) was used to measure urine albumin concentration, according to the instructions provided by the manufacturer.

### Ultrasound imaging of rat kidneys

PCK rats were anesthetized with isoflurane (1.5–2%) in oxygen. Physiological variables (heart rate, respiratory rate, rectal temperature) were continuously monitored by using a VisualSonics Advanced Physiological Monitoring Unit (Toronto, Ontario, Canada). The abdomen was clipped and all hair was removed using Veet hair removal cream. Acoustic coupling was ensured using ultrasound coupling gel. Ultrasound images of the kidneys were acquired using a high resolution ultrasound system (Vevo 2100, VisualSonics) equipped with a 18–38 MHz probe (MS400). Following image optimization, transverse 2D and power Doppler images of both kidneys were acquired with the aid of an automated 3D motor head. Care was taken to include the cranial and caudal poles of the kidney where possible (maximum scan distance 28 mm). The slice thickness between scanning planes was 0.072 mm with a maximum of 500 slices. The images were captured in digital raw format (RF). Offline processing of 3D reconstructions was performed using Vevo 2100 software (v1.6.0) on a dedicated workstation. The resulting 3D model was used to determine the total kidney and cyst volumes and the number of cysts.

### Tissue sectioning, PAS staining, and cystic index determination

For histological examination, one of the kidneys from each rat was sliced perpendicularly to the long axis at approximately 2 mm intervals. Slices from the midportion of the kidneys were fixed in 10% buffered formalin overnight, and tissues were then embedded in paraffin. Three μm sections were stained with periodic acid-Schiff (PAS) following a routine protocol. The stained sections were subjected to cystic index analysis, using the HistoQuest image analysis software (TissueGnostics, Vienna, Austria). The total kidney area (TKA) and the medullary cystic area (MCA) were determined, and the cystic index was calculated in percent as MCA/TKA*100.

### Immunohistochemical analysis

Immunohistochemistry for Ki67 was performed on 3 μm tissue sections which were deparaffinized and rehydrated. Antigen retrieval was performed in an autoclave oven. Mouse anti-Ki67 antibody (BD Pharmingen, San Jose, CA, USA) was applied for 1 hour. Sections were washed and incubated with the biotinylated secondary antibody (Vector Laboratories Inc. Burlingame, CA, USA) for 30 minutes. After washing, the ABC reagent (Vector) was applied to the sections using 3,3’-diaminobenzidine with metal enhancement as the detection reagent. The percentage of Ki-67 positive cells was determined from the total number of cells in cysts and non-cystic tubules from each kidney section. We counted the total number of cells in 10 random fields containing cysts and 10 random fields of non-cystic tissue in each kidney section of each PCK rat using HistoQuest image analysis software (TissueGnostics, Vienna, Austria).

### Determination of cAMP content of whole kidneys

The frozen kidneys were ground to fine powder under liquid nitrogen in a stainless steel mortar. After the liquid nitrogen had evaporated, tissue was weighed and homogenized in 10 volumes of 0.1 M HCl. After centrifugation at 600 g for 10 min at room temperature, the supernatants were collected and assayed for cAMP without acetylation using an enzyme immunoassay kit (Sigma-Aldrich, Inc., St. Louis, MO, USA). The protein content was determined by using the BCA protein assay kit from Pierce (Rockford, Illinois, USA). The results were expressed in pmol/mg of protein.

### Statistics

Data are expressed as means ± SE. The individual parameters of the DAPA group were compared with those of the CON group by a two-tailed Student’s *t*-test for unpaired data by using the GraphPad Prism version 5.0 software (GraphPad, San Diego, CA, USA). *P* values of <0.05 were considered statistically significant.

## Results

### Effect of DAPA treatment on body weight, diuresis and electrolytes

**Fig 1:**
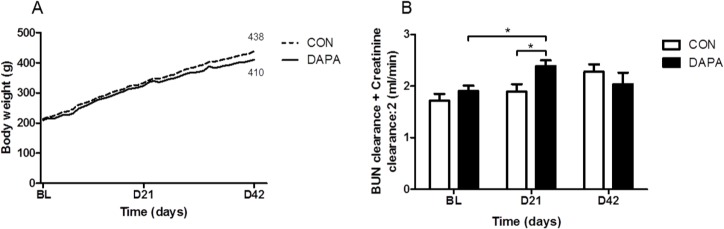
Effect of DAPA on body weight and renal function. Course of body weight in vehicle (CON) and dapagliflozin (DAPA) treated PCK rats during the 6 week treatment phase (A). [Clearance_creatinine_ + Clearance_BUN_ / 2] at baseline (BL) and after 21 and 42 days of treatment in PCK rats (B). N = 8 per group. Data are expressed as means ± SE.* P<0.05.

Treatment with DAPA was well tolerated in PCK rats and they appeared healthy throughout the 6 week treatment phase. Treatment of 6-week old male PCK rats with DAPA (10 mg/kg/d) induced immediate and sustained glucosuria that was accompanied by a 2-fold increase in water intake and a 3-fold increase in urine output when compared with vehicle-treated controls (Table 1). Feces output was also increased by 23% at 3 week and by 45% at 6 week in DAPA-treated PCK rats compared with controls. Plasma glucose (P_glucose_) levels did not change in response to DAPA, and plasma osmolality (P_osm_), sodium (P_Na_^+^) and chloride (P_Cl_^-^) concentrations remained stable at 3 and 6 weeks of treatment. DAPA-treated PCK rats displayed a slightly enhanced urine sodium and chloride excretion rate compared with vehicle-treated rats. Of note, DAPA-treated rats had a 6.4% lower body weight than controls at the end of the 6 week treatment phase (Table 1 and Fig 1A). Treatment with DAPA in normal SD rats also resulted in the induction of glucosuria and an increase in water intake and urine output and a slight increase in urine sodium and chloride, whereas P_Na_^+^ and P_Cl_^-^ remained stable (Table 2).

**Table 1:** Effect of DAPA on body weight, fluid balance and electrolytes in PCK rats.

Time point	Baseline		Day 21		Day 42	
Treatment group	CON	DAPA	CON	DAPA	CON	DAPA
Number of animals	9	9	9	8	9	8
Age, week	6	6	9	9	12	12
Body weight, g	214 ± 5	210 ± 5	333 ± 5	324 ± 6	438 ± 6	410 ± 9*
Water intake, ml/d	31 ± 1	31 ± 1	48 ± 2	92 ± 2***	40 ± 3	86 ± 8***
Diuresis, ml/d	10 ± 1	9 ± 1	18 ± 3	53 ± 2***	19 ± 1	57 ± 7***
P_osm_, mosm/l	294.6 ± 2.2	293.8 ± 1.2	323.0 ± 2.4	315.4 ± 2.9	341.0 ± 7.5	351.6 ± 10.2
P_Na_^+^, mmol/l	137.6 ± 1.6	139.5 ± 0.6	138.5 ± 0.4	140.8 ± 0.4	140.9 ± 0.7	138.8 ± 2.5
P_Cl_^-^, mmol/l	104.8 ± 0.8	105.5 ± 0.3	101.9 ± 0.5	103.9 ± 0.4	105.5 ± 1.6	104.0 ± 1.1
P_glucose_, mmol/l	12.5 ± 1.4	10.6 ± 0.6	11.2 ± 0.4	11.3 ± 0.3	13.4 ±1.3	16.0 ± 2.7
U_osm_, mosm/d	13.9 ± 0.7	14.2 ± 0.7	20.0 ± 2.1	57.2 ± 2.0***	23.9 ±1.0	62.5 ± 5.6***
U_Na_^+^, mmol/d	1.1 ± 0.1	1.1 ± 0.1	1.0 ± 0.1	1.5 ± 0.2*	1.2 ± 0.1	1.8 ± 0.1***
U_Cl_^-^, mmol/d	2.0 ± 0.1	2.1 ±0.1	1.9 ±0.2	2.9 ± 0.2**	2.2 ± 0.1	3.3 ± 0.1***
U_glucose_, mmol/d	0.1 ± 0.0	0.0 ± 0.0	0.3 ± 0.2	24.6 ± 0.9***	0.3 ± 0.1	23.6 ± 4.3***

Data are expressed as means ± SE. CON, vehicle control; DAPA, dapagliflozin.

*P<0.05

**P<0.01

***P<0.001, when comparing DAPA and CON at each time point. P, plasma; U, urine.

**Table 2:** Effect of DAPA on body weight, fluid balance and electrolytes in normal SD rats.

Time point	Baseline		Day 18		Day 35	
Treatment group	CON	DAPA	CON	DAPA	CON	DAPA
Number of animals	9	9	9	9	9	8
Age, week	5	5	7.5	7.5	10	10
Body weight, g	144 ± 2	149 ± 7	285 ± 5	297 ± 7	349 ± 5	354 ± 6
Water intake, ml/d	32 ± 1	32 ± 3	41 ± 2	54 ± 2***	49 ± 3	62 ± 7*
Diuresis, ml/d	11 ± 1	12 ± 1	17 ± 2	24 ± 3*	32 ± 2	36 ± 4
P_Na_^+^, mmol/l	138.9 ± 0.4	142.3 ± 3.4	138.4 ± 0.4	133.5 ± 1.6*	141.9 ± 0.5	140.4 ± 0.6
P_Cl_^-^, mmol/l	100.9 ± 0.3	100.6 ± 1.0	100.3 ± 0.3	94.0 ± 3.0	100.9 ± 0.3	95.9 ± 2.3
P_glucose_, mmol/l	10.5 ± 0.6	10.3 ± 0.2	9.9 ± 0.2	9.7 ± 0.3	13.4 ± 0.2	13.0 ± 0.5
U_Na_^+^, mmol/d	1.3 ± 0.1	1.2 ± 0.1	1.1 ± 0.1	1.4 ± 0.1	1.1 ± 0.3	1.6 ± 2.0
U_Cl_^-^, mmol/d	2.1 ± 0.1	1.8 ± 0.2	1.8 ± 0.1	2.1 ± 0.1	2.3 ± 0.1	2.4 ± 0.5
U_glucose_, mmol/d	0.0 ± 0.0	0.0 ± 0.0	0.0 ± 0.0	10.6 ± 0.6***	0.0 ± 0.0	13.5 ± 2.0***

Data are expressed as means ± SE. CON, vehicle control; DAPA, dapagliflozin.

*P<0.05

**P<0.01

***P<0.001, when comparing DAPA and CON at each time point. P, plasma; U, urine.

### Effect of DAPA treatment on renal function and albuminuria

Table 3 shows that the plasma creatinine concentrations were similar at 3 weeks but tended to increase at 6 weeks in DAPA-treated PCK rats. The plasma BUN concentrations were significantly higher at 3 and 6 weeks of DAPA treatment. The clearances of creatinine and BUN were significantly higher at 3 weeks, but the difference disappeared at 6 weeks of treatment with DAPA. Calculating the [clearance_creatinine_ + clearance_BUN_ / 2] confirmed that there was a higher clearance at 3 but not at 6 weeks of DAPA treatment in PCK rats (Fig 1B). Table 4 shows the data in normal SD rats. In DAPA-treated normal rats we did not see an increase in the creatinine and BUN clearances, and the [clearance_creatinine_ + clearance_BUN_ / 2] was also similar between DAPA- vs. vehicle-treated SD rats. These data suggest that there was transient hyperfiltration and subsequent deterioration of renal function upon treatment with DAPA in PCK but not in SD rats.

**Table 3:** Effect of DAPA on renal function in PCK rats.

Time point	Baseline		Day 21		Day 42	
Treatment group	CON	DAPA	CON	DAPA	CON	DAPA
Number of animals	9	9	9	8	9	8
Age, week	6	6	9	9	12	12
P_creatinine_, μmol/l	18.0 ± 0.0	18.0 ± 0.0	21.9 ±0.5	21.4 ±0.5	26.9 ± 1.2	36.1 ± 6.7
Clearance_creatinine_, ml/min	2.4 ± 0.2	2.7 ± 0.2	2.6 ± 0.2	3.1 ±0.1*	3.2 ± 0.2	2.7 ± 0.3
P_BUN_, mmol/l	3.7 ± 0.1	3.9 ±0.1	4.1 ± 0.2	4.9 ± 0.2*	5.7 ± 0.1	7.1 ± 0.6*
Clearance_BUN_, ml/min	1.0 ± 0.1	1.1 ± 0.1	1.2 ±0.1	1.7 ± 0.1**	1.3 ± 0.1	1.4 ± 0.1
[Clearance_creatinine_ + Clearance_BUN_ / 2]	1.7 ± 0.1	1.9 ± 0.1	1.8 ± 0.1	2.3 ± 0.1*	2.2 ± 0.1	2.0 ± 0.1

Data are expressed as means ± SE. CON, vehicle control; DAPA, dapagliflozin.

*P<0.05

**P<0.01, when comparing CON and DAPA at each time point. P, plasma.

**Table 4:** Effect of DAPA on renal function in normal SD rats.

Time point	Baseline		Day 18		Day 35	
Treatment group	CON	DAPA	CON	DAPA	CON	DAPA
Number of animals	9	9	9	9	9	8
Age, week	5	5	7.5	7.5	10	10
P_creatinine_, μmol/l	26.5 ± 0.0	26.5 ± 0.1	26.5 ± 0.0	26.5 ± 0.2	35.3 ± 2.5	35.3 ± 3.2
Clearance_creatinine_, ml/min	1.7 ± 0.1	1.2 ± 0.3	2.0 ± 0.1	2.0 ± 0.2	2.6 ± 0.1	2.7 ± 0.2
P_BUN_, mmol/l	6.6 ± 0.3	5.5 ± 0.2	6.1 ± 0.2	8.2 ± 0.4***	6.6 ± 0.1	9.9 ± 1.5*
Clearance_BUN_, ml/min	0.6 ± 0.0	0.8 ± 0.1	0.8 ± 0.0	0.8 ± 0.1	1.1 ± 0.0	0.8 ± 0.2*
[Clearance_creatinine_ + Clearance_BUN_ / 2]	1.1 ± 0.1	1.0 ± 0.2	1.4 ± 0.1	1.2 ± 0.2	1.9 ± 0.1	1.9 ± 0.1

Data are expressed as means ± SE. CON, vehicle control; DAPA, dapagliflozin.

*P<0.05

**P<0.01

***P<0.001 when comparing CON and DAPA at each time point. P, plasma.

**Fig 2:**
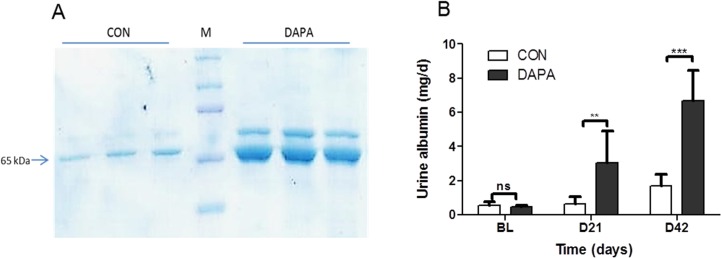
Effect of DAPA on albumin excretion. Urine protein analysis by SDS-PAGE from urine samples of PCK rats after 6 weeks of treatment with vehicle (CON) or dapagliflozin (DAPA). Each lane shows urine sample of a single PCK rat. Sample volumes were corrected for diuresis and amounted to 15 μl for CON and 45 μl for DAPA. Arrow shows the band for albumin. M = molecular weight marker (A). Urine albumin concentration as measured by ELISA, in mg/day (B). N = 8 per group. Columns represent means ± SE. **P<0.01, ***P<0.001 when comparing CON and DAPA at each time point, ns = non-significant.

Since albuminuria correlates with disease severity in PKD we analyzed the urine protein content by SDS-PAGE and determined the urine albumin excretion by ELISA (Fig 2). SDS-PAGE of urines (adjusted for 24 h urine volumes) revealed a marked increase of albuminuria in DAPA-treated PCK at 6 weeks (Fig 2A). The ELISA showed that albumin excretion increased slightly at 3 weeks and a further increase was observed at 6 weeks in vehicle-treated PCK rats, as expected (Fig 2B). In DAPA-treated rats the albuminuria increased markedly at 3 weeks and more importantly at 6 weeks. After 6 weeks the albumin excretion amounted to 1.7 ± 0.3 mg/d in vehicle-treated rats and to 6.8 ± 0.7 mg/day in DAPA-treated animals (*P*<0.0001). In normal SD rats, the excretion of urine albumin was lower than in PCK rats but also increased slightly upon treatment with DAPA for 5 weeks (0.4 ± 0.0 vs. 1.2 ± 0.2 mg/day, *P*<0.05).

### *In vivo* high resolution ultrasound imaging of rat kidneys

**Fig 3:**
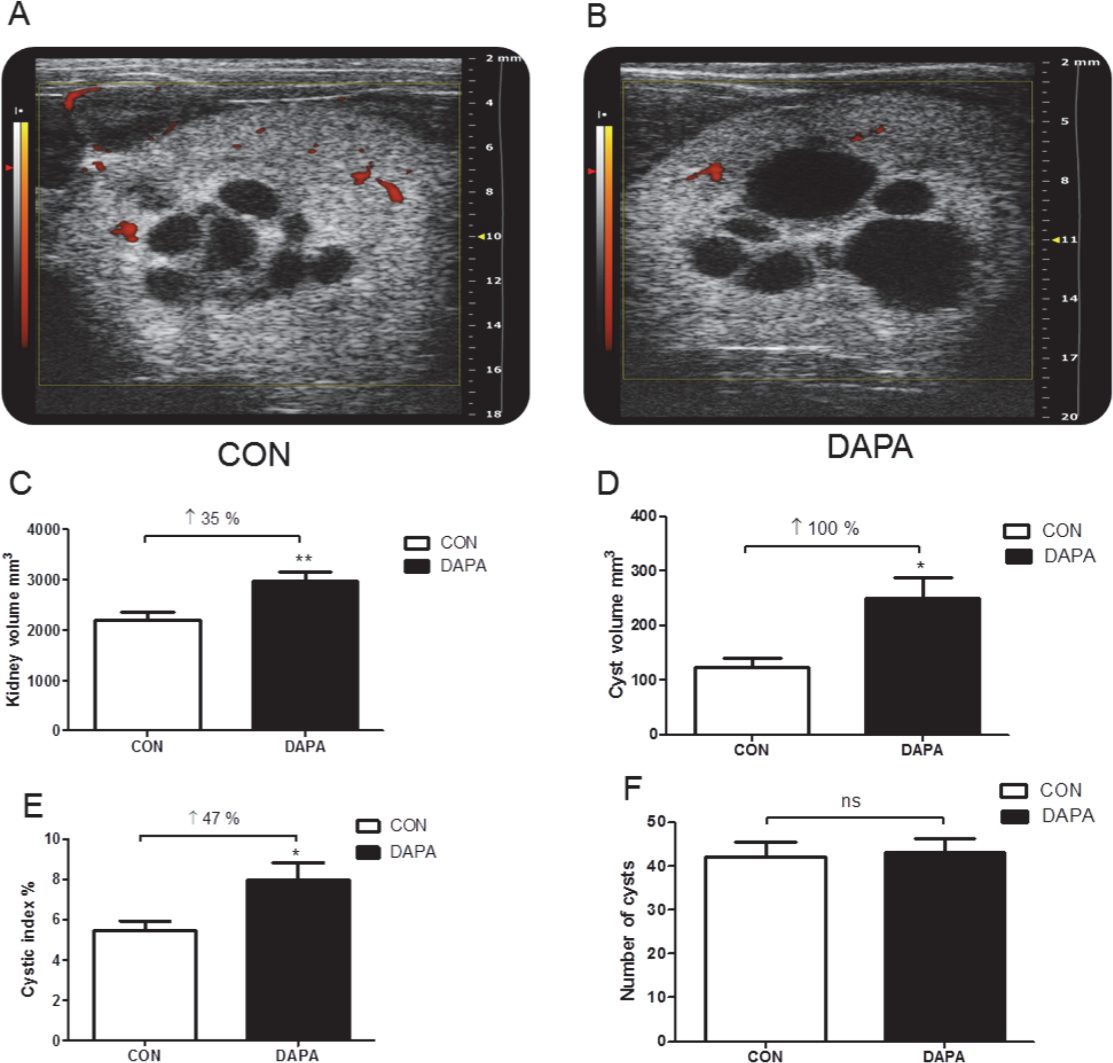
Effect of DAPA on kidney volume and cystic index. Typical 2D transverse power Doppler ultrasound images of a kidney in CON- and DAPA-treated PCK rats. The red areas represent a power Doppler signal consistent with blood flow within the renal vasculature. The maximum scan distance between cranial and caudal poles of the kidney was 28 mm. The slice thickness between scanning planes was 0.072 mm with a maximum of 500 slices (A, B). Change in kidney volume (C), cyst volume (D), cystic index (E) and number of cysts (F) in PCK rats. N = 6 rats or 12 kidneys per group. Columns represent means ± SE. *P<0.05, **P<0.01, ns = non-significant.

After 6 weeks of treatment, the PCK rats were anesthetized for ultrasound analysis of both kidneys. Fig 3A shows that the kidneys with the typical medullary cysts could be easily visualized in vehicle-treated male PCK rats. On the other hand, DAPA-treated rats displayed larger kidneys and had substantially bigger cysts (Fig 3B). Ultrasound-based measurements revealed a 35% higher total kidney volume (*P*<0.01), a 2-fold higher cyst volume (*P*<0.05) and a 47% higher cystic index (*P*<0.05) in DAPA- vs. vehicle-treated rats, whereas the number of cysts was similar in both groups after 6 weeks of treatment (*P* = 0.841) (Fig 3C–3F).

### Effect of DAPA treatment on kidney weight and morphology

**Fig 4:**
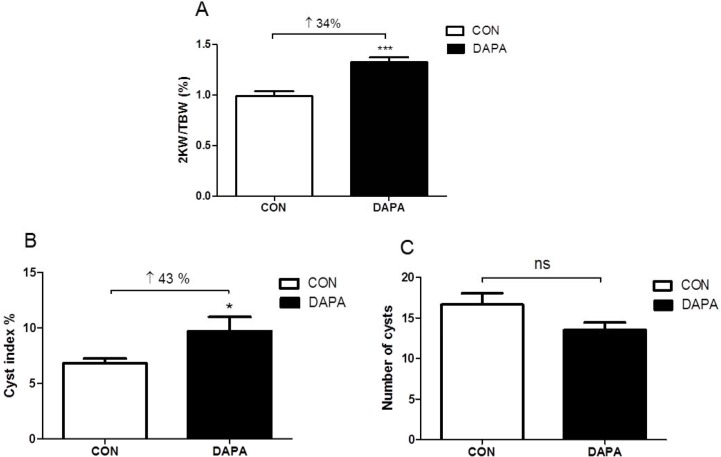
Effect of DAPA on kidney weight and cyst index. Ratio of two kidney weights to total body weight (2KW/TBW) (A), cyst index (B) and number of cysts (C) after 6 week treatment with vehicle (CON) or DAPA in PCK rats. N = 8 per group. Columns represent means ± SE. *P<0.05, ***P<0.001, ns = non-significant.

**Fig 5:**
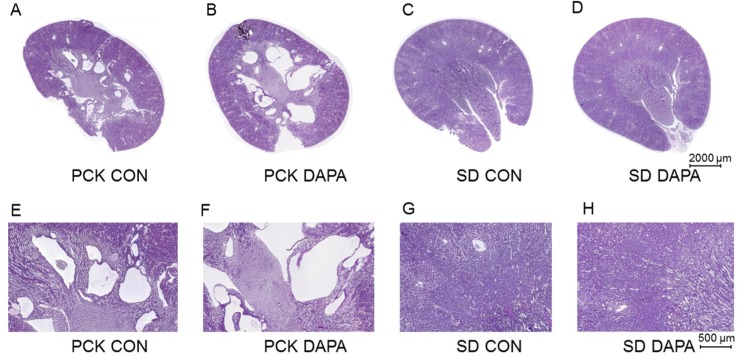
Effect of DAPA on renal histology. Representative renal histology by periodic acid-Schiff (PAS) staining of kidneys in vehicle- treated (CON) PCK (A,E) and normal SD (C,G) rats and in DAPA-treated PCK (B,F) and normal SD (D,H) rats. Scale bar is 2000 μm in A and B and 500 μm in C and D.

After 6 weeks of treatment with DAPA the total weight of both kidneys was 23% higher in DAPA- as compared with vehicle-treated male PCK rats (4.32 ± 0.24 vs. 5.33 ± 0.20 g, *P<*0.01). The two kidneys to body weight (2K/BW) ratio was also significantly increased by 34% in the DAPA-treated rats (*P*<0.001). PAS-staining of kidney sections revealed that the cyst index was increased by 43% in DAPA-treated rats (*P*<0.05) while the total cyst number was not significantly changed (*P* = 0.074) (Figs 4 and 5). In normal SD rats the total kidney weight of both kidneys was also higher in DAPA-treated rats as compared to the total kidney weight in vehicle-treated rats (3.1± 0.3 vs. 2.3 ± 0.2 g, *P* = 0.0002).

### Effect of DAPA treatment on renal cAMP content and renal epithelial cell proliferation

**Fig 6:**
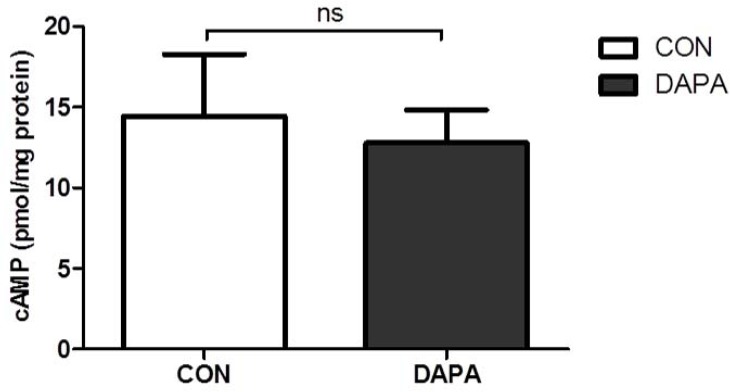
Effect of DAPA on renal cAMP content. Analysis of cAMP concentration per mg protein in PCK rat kidneys treated with vehicle (CON) or dapagliflozin (DAPA). N = 8 per group. Columns represent means ± SE. ns = non-significant.

Since cAMP is a major driver of cyst growth we analyzed the content of cAMP in PCK kidney tissue by ELISA. Fig 6 shows that the amount of cAMP per mg of total protein was similar in DAPA- and vehicle-treated male PCK rats (*P* = 0.717).

**Fig 7:**
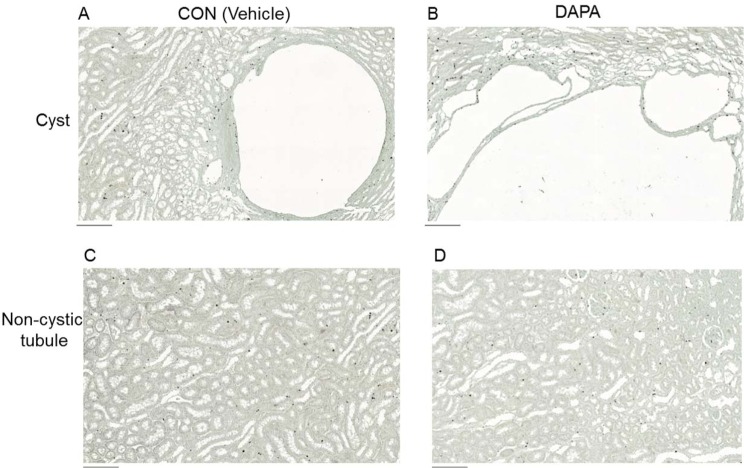
Effect of DAPA on cell proliferation. Representative areas of Ki67 immunohistochemical staining in PCK rat kidneys are shown. Nuclei of Ki67-positive cells were stained brown with 3,3’-diaminobenzidine and that of Ki67-negative cells appeared blue because of the counterstaining with hematoxylin. CON (cystic area) (A), DAPA (cystic area) (B), CON (non-cystic area) (C), DAPA (non-cystic area) (D). Scale bar is 200 μm in A, B, C and D.

Immunohistochemistry staining for Ki67 was then used to examine whether DAPA treatment had an effect on tubular and cystic epithelial cell proliferation. Fig 7 shows no significant change in the number of Ki67-positive nuclei in the cystic epithelium in DAPA-treated PCK rats. Quantification of the Ki67-positive nuclei confirmed that the number of Ki67-positive nuclei was not changed in cystic epithelium as well as in non-cystic epithelium in DAPA- vs. vehicle-treated PCK rats (Table 5).

**Table 5:** Effect of DAPA on cell proliferation in PCK rat kidneys.

Treatment	CON	DAPA	Difference, %	*P*-value
Number of animals	7	7		
Ki67-positive cells (%) in cyst-lining epithelium	6.5 ± 1.3	6.7 ± 1.9	+ 3.5	0.922
Ki67-positive cells (%) in non-cystic tubules	2.5 ± 0.91	3.1 ± 1.1	+ 24.7	0.677

Morphometric analysis using Ki67, a proliferation marker in kidney medulla of vehicle (CON)- and DAPA-treated PCK rats. Data are expressed as means ± SE.

## Discussion

Here we show that the induction of osmotic diuresis with the SGLT2-specific inhibitor DAPA leads to an unexpected increase in the kidney and renal cyst volumes of PCK rats, an orthologous model of ARPKD. Furthermore, DAPA-treated PCK but not normal SD rats displayed a transient increase in the clearances of creatinine and BUN and a progressive increase in albuminuria which suggests that DAPA promotes hyperfiltration in PCK rats. The renal cAMP content was similar, and there was no evidence for heightened epithelial cell proliferation which could explain the greater cyst volume upon treatment with DAPA. Thus, the induction of glucosuria with DAPA appeared to have a negative impact on cystic disease progression in PCK rats.

The increase in glomerular filtration in response to DAPA appeared to be transient, i.e. it was seen after 3 weeks but not after 6 weeks. However it was associated with an increase in albuminuria at 3 weeks and a further increase at 6 weeks. In general, an increase in albuminuria is seen with progressing stages of PKD [20]. The increased amount of urine albumin could be the consequence of enhanced leakage of albumin from glomeruli, or alternatively it could be caused by decreased tubular reabsorption. Whether albuminuria is reflecting glomerular hyperfiltration damage needs to be examined further.

Treatment with SGLT2 inhibitors has been associated with a decrease in the GFR in normal and diabetic rats and in humans with type 2 diabetes mellitus [21,22]. This contrasts with our findings in PCK rats which display evidence of transient hyperfiltration. This hyperfiltration could in part be explained by the enhanced food i.e. protein intake which is seen in response to DAPA [23], since protein loading is a known stimulus for increasing glomerular filtration. However this should have been counterbalanced by the higher sodium delivery at the macula densa which normally leads to afferent arteriolar vasoconstriction [24]. Since normal rats which are treated with DAPA do not develop hyperfiltration and albuminuria [25–27], the effects that we observed appear to be specific to the PCK model of PKD.

In a previous study, we found that treatment with the SGLT1/SGLT2 inhibitor phlorizin attenuated albuminuria in Han:SPRD rats, improved GFR, and decreased cyst growth [18]. Contrasting with these results, the DAPA-treated PCK rats displayed enhanced cyst growth. Han:SPRD rats are a non-orthologous model of PKD, with cyst formation exclusively in the proximal tubules [28,29]. In contrast, PCK rats display cyst formation in the distal nephron. Typically, the cysts disconnect from the original proximal tubule in Han:SPRD, whereas in PCK rats the cysts remain connected to the distal tubule. We speculate that the increased intratubular osmotic pressure which is caused by the glucosuria could promote the dilation of distal tubular segments and cysts. This could cause compression of adjacent healthy distal tubules and provide a stimulus to enhance GFR, as it may be seen in partial obstructive uropathy [30].

The enhanced cyst growth did not appear to be the consequence of vasopressin-mediated cAMP stimulation, since the renal cAMP content was similar between DAPA- and vehicle-treated PCK rats. Despite a massive increase in the diuresis, DAPA-treated rats did not appear to be dehydrated, and the serum sodium concentrations did not increase. This suggests indirectly that vasopressin levels did not increase. Furthermore, Ki67 staining did not reveal an increase in proliferating cells in dilated tubules, and there was no change in the number of nuclei in the cysts upon DAPA treatment. This indicates again that the cysts increased in size due to osmotic pressure without affecting epithelial cell proliferation.

Diabetes may occur in patients with ADPKD and it is associated with greater cardiovascular morbidity [31]. With the possibility to treat ADPKD patients with SGLT2 inhibitors there could be a risk of albuminuria and increased cyst growth. Although such a complication has not yet been described caution should be exerted when prescribing these drugs to patients with ADPKD.

In summary, we have shown that the induction of osmotic diuresis by inhibiting SGLT2 promotes transient hyperfiltration, albuminuria and increased cyst volume in PCK rats. Further studies need to explore the mechanisms which link the enhanced glomerular filtration to the increase in cyst volume upon SGLT inhibition in this model of PKD.

## References

[1] Harris PC, Torres VE (2009) Polycystic kidney disease. Annu Rev Med 60: 321–337. 10.1146/annurev.med.60.101707.125712 18947299 PMC2834200

[2] Torres VE, Harris PC, Pirson Y (2007) Autosomal dominant polycystic kidney disease. Lancet 369: 1287–1301. 17434405 10.1016/S0140-6736(07)60601-1

[3] Schrier RW, McFann KK, Johnson AM (2003) Epidemiological study of kidney survival in autosomal dominant polycystic kidney disease. Kidney Int 63: 678–685. 12631134 10.1046/j.1523-1755.2003.00776.x

[4] Wüthrich RP, Mei C (2014) Pharmacological management of polycystic kidney disease. Expert Opin Pharmacother 15: 1085–1095. 10.1517/14656566.2014.903923 24673552

[5] Devuyst O, Torres VE (2013) Osmoregulation, vasopressin, and cAMP signaling in autosomal dominant polycystic kidney disease. Curr Opin Nephrol Hypertens 22: 459–470. 10.1097/MNH.0b013e3283621510 23736843

[6] Torres VE, Harris PC (2014) Strategies targeting cAMP signaling in the treatment of polycystic kidney disease. J Am Soc Nephrol 25: 18–32. 10.1681/ASN.2013040398 24335972 PMC3871779

[7] Meijer E, Gansevoort RT, de Jong PE, van der Wal AM, Leonhard WN, de Krey SR, et al (2011) Therapeutic potential of vasopressin V2 receptor antagonist in a mouse model for autosomal dominant polycystic kidney disease: optimal timing and dosing of the drug. Nephrol Dial Transplant 26: 2445–2453. 10.1093/ndt/gfr069 21393612

[8] Gattone VH 2nd, Wang X, Harris PC, Torres VE (2003) Inhibition of renal cystic disease development and progression by a vasopressin V2 receptor antagonist. Nat Med 9: 1323–1326. 14502283 10.1038/nm935

[9] Torres VE, Wang X, Qian Q, Somlo S, Harris PC, Gattone VH 2nd (2004) Effective treatment of an orthologous model of autosomal dominant polycystic kidney disease. Nat Med 10: 363–364. 14991049 10.1038/nm1004

[10] Wang X, Gattone V 2nd, Harris PC, Torres VE (2005) Effectiveness of vasopressin V2 receptor antagonists OPC-31260 and OPC-41061 on polycystic kidney disease development in the PCK rat. J Am Soc Nephrol 16: 846–851. 15728778 10.1681/ASN.2004121090

[11] Hopp K, Wang X, Ye H, Irazabal MV, Harris PC, Torres VE (2015) Effects of hydration in rats and mice with polycystic kidney disease. American Journal of Physiology—Renal Physiology 308: F261–F266. 25503729 10.1152/ajprenal.00345.2014 PMC4312959

[12] Torres VE, Chapman AB, Devuyst O, Gansevoort RT, Grantham JJ, Higashihara E, et al (2012) Tolvaptan in patients with autosomal dominant polycystic kidney disease. N Engl J Med 367: 2407–2418. 10.1056/NEJMoa1205511 23121377 PMC3760207

[13] Wang X, Wu Y, Ward CJ, Harris PC, Torres VE (2008) Vasopressin directly regulates cyst growth in polycystic kidney disease. J Am Soc Nephrol 19: 102–108. 18032793 10.1681/ASN.2007060688 PMC2391034

[14] Nagao S, Nishii K, Katsuyama M, Kurahashi H, Marunouchi T, Takahashi H, et al (2006) Increased water intake decreases progression of polycystic kidney disease in the PCK rat. J Am Soc Nephrol 17: 2220–2227. 16807403 10.1681/ASN.2006030251

[15] Ho TA, Godefroid N, Gruzon D, Haymann JP, Marechal C, Wang X, et al (2012) Autosomal dominant polycystic kidney disease is associated with central and nephrogenic defects in osmoregulation. Kidney Int 82: 1121–1129. 10.1038/ki.2012.225 22718190

[16] Torres VE, Bankir L, Grantham JJ (2009) A case for water in the treatment of polycystic kidney disease. Clin J Am Soc Nephrol 4: 1140–1150. 10.2215/CJN.00790209 19443627

[17] Wang CJ, Creed C, Winklhofer FT, Grantham JJ (2011) Water prescription in autosomal dominant polycystic kidney disease: a pilot study. Clin J Am Soc Nephrol 6: 192–197. 10.2215/CJN.03950510 20876670 PMC3022242

[18] Wang X, Zhang S, Liu Y, Spichtig D, Kapoor S, Koepsell H, et al (2013) Targeting of sodium-glucose cotransporters with phlorizin inhibits polycystic kidney disease progression in Han:SPRD rats. Kidney Int 84: 962–968. 10.1038/ki.2013.199 23715121

[19] Mather A, Pollock C (2010) Renal glucose transporters: novel targets for hyperglycemia management. Nat Rev Nephrol 6: 307–311. 10.1038/nrneph.2010.38 20351704

[20] Chapman AB, Johnson AM, Gabow PA, Schrier RW (1994) Overt proteinuria and microalbuminuria in autosomal dominant polycystic kidney disease. J Am Soc Nephrol 5: 1349–1354. 7894001 10.1681/ASN.V561349

[21] Skrtic M, Yang GK, Perkins BA, Soleymanlou N, Lytvyn Y, von Eynatten M, et al (2014) Characterisation of glomerular haemodynamic responses to SGLT2 inhibition in patients with type 1 diabetes and renal hyperfiltration. Diabetologia 57: 2599–2602. 10.1007/s00125-014-3396-4 25280671

[22] De Nicola L, Gabbai FB, Liberti ME, Sagliocca A, Conte G, Minutolo R (2014) Sodium/glucose cotransporter 2 inhibitors and prevention of diabetic nephropathy: targeting the renal tubule in diabetes. Am J Kidney Dis 64: 16–24. 10.1053/j.ajkd.2014.02.010 24673844

[23] Devenny JJ, Godonis HE, Harvey SJ, Rooney S, Cullen MJ, Pelleymounter MA (2012) Weight loss induced by chronic dapagliflozin treatment is attenuated by compensatory hyperphagia in diet-induced obese (DIO) rats. Obesity (Silver Spring) 20: 1645–1652. 10.1038/oby.2012.59 22402735

[24] Thomson SC, Rieg T, Miracle C, Mansoury H, Whaley J, Vallon V, et al (2012) Acute and chronic effects of SGLT2 blockade on glomerular and tubular function in the early diabetic rat. Am J Physiol Regul Integr Comp Physiol 302: R75–83. 10.1152/ajpregu.00357.2011 21940401 PMC3349378

[25] Meng W, Ellsworth BA, Nirschl AA, McCann PJ, Patel M, Girotra RN, et al (2008) Discovery of dapagliflozin: a potent, selective renal sodium-dependent glucose cotransporter 2 (SGLT2) inhibitor for the treatment of type 2 diabetes. J Med Chem 51: 1145–1149. 10.1021/jm701272q 18260618

[26] Han S, Hagan DL, Taylor JR, Xin L, Meng W, Biller SA, et al (2008) Dapagliflozin, a selective SGLT2 inhibitor, improves glucose homeostasis in normal and diabetic rats. Diabetes 57: 1723–1729. 10.2337/db07-1472 18356408

[27] Obermeier M, Yao M, Khanna A, Koplowitz B, Zhu M, Li W, et al (2010) In vitro characterization and pharmacokinetics of dapagliflozin (BMS-512148), a potent sodium-glucose cotransporter type II inhibitor, in animals and humans. Drug Metab Dispos 38: 405–414. 10.1124/dmd.109.029165 19996149

[28] Brown JH, Bihoreau MT, Hoffmann S, Kranzlin B, Tychinskaya I, Obermuller N, et al (2005) Missense mutation in sterile alpha motif of novel protein SamCystin is associated with polycystic kidney disease in Cy/+ rat. J Am Soc Nephrol 16: 3517–3526. 16207829 10.1681/ASN.2005060601

[29] Hoff S, Halbritter J, Epting D, Frank V, Nguyen TM, van Reeuwijk J, et al (2013) ANKS6 is a central component of a nephronophthisis module linking NEK8 to INVS and NPHP3. Nat Genet 45: 951–956. 10.1038/ng.2681 23793029 PMC3786259

[30] Chevalier RL (1998) Pathophysiology of obstructive nephropathy in the newborn. Semin Nephrol 18: 585–593. 9819149

[31] Reed B, Helal I, McFann K, Wang W, Yan XD, Schrier RW (2012) The impact of type II diabetes mellitus in patients with autosomal dominant polycystic kidney disease. Nephrol Dial Transplant 27: 2862–2865. 10.1093/ndt/gfr744 22207329 PMC3398061

